# Association between serum copper and COPD: Insights from NHANES 2011–2016 and Mendelian randomization study

**DOI:** 10.18332/tid/210412

**Published:** 2025-10-17

**Authors:** Jiajia Qu, Mengyu Zhang, Chenyang Hu, Yongli Liu, Wei Zhao, Yiqing Qu

**Affiliations:** 1 Department of Pulmonary and Critical Care Medicine, Qilu Hospital, Shandong University, Jinan, China

**Keywords:** chronic obstructive pulmonary disease, mendelian randomization, serum copper, GWAS, NHANES

## Abstract

**INTRODUCTION:**

Chronic obstructive pulmonary disease (COPD) affects 390 million people globally, with oxidative stress playing a key pathogenic role. Smoking and other forms of tobacco exposure are major COPD drivers and important sources of systemic oxidative stress, and potentially interact with metal homeostasis. Copper exhibits dual effects in lung homeostasis, as a cofactor for antioxidant enzymes and a potential catalyst for reactive oxygen species. However, the causal relationship between serum copper levels and COPD remains unclear. This study aimed to assess their association using a combination of observational and genetic approaches.

**METHODS:**

In stage one, we used multivariate regression to analyze the association between serum copper and COPD in 3166 participants in the National Health and Nutrition Examination Survey (NHANES), 2011–2016. Models adjusted for demographic and clinical covariates including smoking-related variables, and stratified analyses by smoking status. Stage two utilized Mendelian randomization (MR) analysis to explore a potential cause-and-effect link between copper levels in serum determined by genetics and COPD.

**RESULTS:**

Observational analysis showed increased COPD risk in the highest serum copper tertile (T3 vs T1, OR=1.65; 95% CI: 1.09–2.49; p-trend=0.0245). The association remained after adjustment for smoking-related covariates. However, MR analyses using both FinnGen and UK Biobank data suggested no causal effect (FinnGen IVW, OR=1.01; 95% CI: 0.98–1.04, p=0.37; UK Biobank IVW, OR=1.00; 95% CI: 1.00–1.00, p=0.55), with sensitivity analyses confirming result robustness.

**CONCLUSIONS:**

While elevated serum copper is associated with COPD prevalence observationally, the null MR finding suggests it may reflect tobacco-related systemic oxidative stress or reverse causation rather than being a direct causal driver. Therefore, serum copper may be more useful as a biomarker of smoking-induced redox disturbance than as a therapeutic target. These results underscore the importance of integrating tobacco exposure metrics in future studies examining metal biology in COPD.

## INTRODUCTION

Chronic obstructive pulmonary disease (COPD) is characterized by airways and alveoli abnormalities leading to persistent airflow limitation with progressive clinical deterioration^1^. According to the World Health Organization (WHO), COPD is currently recognized as the third leading global cause of mortality, reflecting its substantial disease burden and high fatality rates^2^. Increasing evidence supports that the central mechanisms driving COPD include aging and heightened oxidative stress, processes that are strongly amplified by tobacco exposure^3,4^.

Maintaining metal ion homeostasis is crucial for pulmonary redox balance^5^. While iron and zinc have been extensively studied in COPD, the role of copper remains contentious^6,7^. As a cofactor for antioxidant enzymes like superoxide dismutase (SOD), copper paradoxically catalyzes ROS generation via Fenton reactions^8^. This dual role is reflected in conflicting evidence: in vivo studies demonstrate copper deficiency induces emphysema in mice through impaired antioxidant defenses^9,10^, whereas epidemiological studies associate elevated serum copper with COPD risk, potentially reflecting compensatory responses to systemic oxidative stress^11,12^. Notably, tobacco use is a major source of systemic and pulmonary oxidative burden and may both modify copper metabolism and confound observational associations; thus, explicitly considering smoking-related measures is essential when evaluating copper-COPD links. The results underscore the intricate link between copper concentrations and COPD, necessitating additional research.

Conventional observational studies face inherent limitations – serum copper levels are influenced by smoking, diet, and unrecognized comorbidities, complicating interpretation of causal effects^13,14^. Here, we integrate observational study with Mendelian randomization (MR), a genetic instrumental variable approach that strongly suggests potential causal associations by leveraging lifelong exposure levels predicted by copper-related SNPs. This study therefore aimed to assess the correlation between serum copper and COPD using a dual methodology, distinguishing whether copper dysregulation may contribute to COPD pathogenesis or merely accompanies systemic oxidative stress and other smoking-related processes.

## METHODS

### Study design

**Figure 1:**
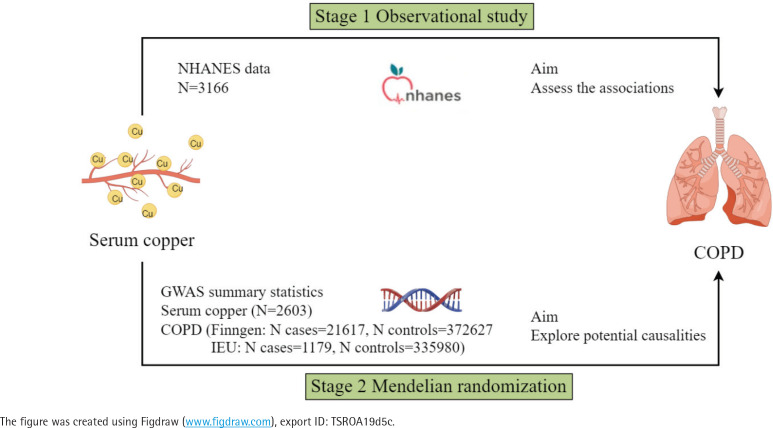
Overall study design of the present study. Stage 1: An observational study was conducted using NHANES data (2011–2016, N=3166) to assess the associations between serum copper levels and COPD. Stage 2: Mendelian randomization analysis was performed using GWAS summary statistics for serum copper (N=2603) and COPD (FinnGen: 21617 cases and 372627 controls; IEU: 1179 cases and 335980 controls) to explore potential causal relationships

As depicted in Figure 1, the research unfolded in a two-stage process. In the early stage, we conducted a multivariate regression analysis using the NHANES database^15^ to investigate the link between serum copper concentrations and COPD. In the following phase, MR analysis assessed the causal relationship between genetic serum copper concentrations and COPD, utilizing Genome-Wide Association Study (GWAS) summary data.

### Data acquisition

The objective of the NHANES survey is to evaluate the US population’s health and nutrition via a comprehensive, layered, multi-phase national survey^16^. Adhering to the Declaration of Helsinki, the research utilized NHANES data accessible to the public from 2011 to 2016.

The exclusion criteria were as: 1) aged <20 years; 2) absence of COPD data; 3) absence of serum copper measurements; and 4) missing covariate data. The flow chart with information on exclusion criteria and characteristics of participants excluded are presented in Supplementary file Figure 1. In the end, 3166 respondents – 2910 controls and 256 COPD patients – were included in the research.

### COPD definition

Previous studies^17-19^ have identified COPD as: 1) a ratio of forced expiratory volume in one second (FEV1) to forced vital capacity (FVC) <70% for 2007–2012; and 2) self-reported through three distinct questionnaires between 2013 and 2018, owing to insufficient spirometry data, asking: ‘Has your doctor ever informed you about your chronic bronchitis?’, ‘Has a doctor ever told you that you have emphysema?’ as well as ‘Has a doctor or other health professional ever told you that you have COPD?’. Individuals who responded ‘yes’ to any of these three queries were categorized as members of the COPD group; if they selected ‘no’ for all three questions, they were classified as belonging to the non-COPD group.

### Serum copper measurement

The measurement of copper in serum was conducted through inductively coupled plasma dynamic reaction mass spectrometry (ICP-DRC-MS), with comprehensive guidelines found in the Laboratory Procedures Manual^15^. Levels of copper in the serum (μg/dL) were classified into three distinct categories: low, medium, and high.

### Assessment of covariates

Factors considered were age (20–39, 40–59, ≥60 years), gender (male, female), ethnicity (Mexican-American, non-Hispanic White, non-Hispanic Black, Other), education level (lower than high school, high school, higher than high school), and poverty-to-income ratio (PIR). PIR was calculated as the ratio of self-reported family income ($) to the federal poverty threshold, which is determined annually by the US Department of Health and Human Services^20^. Participants were categorized into three groups: <1.40, 1.40–3.49, and >3.49. Other variables included BMI (<25, 25–29.9, ≥30 kg/m^2^), heart disease, high blood pressure, diabetes, and smoking habits. The definition of cardiovascular disease (CVD) for respondents was: ‘Has a doctor or health professional ever informed you of having congestive heart failure (CHF), coronary heart disease (CHD), angina, heart attack, or stroke?’. Hypertension is characterized by either systolic or diastolic blood pressure levels of 130/80 mmHg or higher, or by self-reported diagnosis or the utilization of antihypertensive medications. Diabetic signs encompassed a 2-hour oral glucose tolerance test indicating ≥11.1 mmol/L, fasting glucose levels of ≥7 mmol/L, random glucose readings of ≥11 mmol/L, glycosylated hemoglobin levels of ≥6.5%, and self-reported use of diabetes or antidiabetic drugs. The classification of smoking status was segmented into current, former, or never: current smokers consumed more than 100 cigarettes, former smokers over 100 but quit, and never smokers had no more than 100 cigarettes in their life.

### Mendelian randomization

Our assessment focused on evaluating the potential causal relationship between serum copper levels and COPD, employing a two-sample MR method. Supplementary file Figure 2 illustrates the fundamental concepts of MR along with three presuppositions. The three presumptions are: 1) genetic variations must be linked to serum copper levels; 2) they should not be linked to confounding factors; and 3) they should only be the mechanism by which serum copper levels mediate COPD. Genetic instruments for serum copper were obtained from the IEU OpenGWAS project (ieu-a-1073)^21^, which included 2603 individuals of European ancestry. COPD data were sourced from the Finngen^22^ and UK Biobank^23^ (Finngen_R11_J10_COPD and ukb-a-67). Detailed information on exposure and outcome data can be obtained from Supplementary file Table 1. SNPs were selected based on the criteria: Ld r^2^ <0.001, kb=10000, and p<5×10^-6^.

### Statistical analysis

The weights assigned to the samples adhered to the NHANES analytical standards^24^. The demographic details of the classified variables were denoted as n (weighted percentage), and variances were evaluated through Fisher’s exact test or the chi-squared test. The link between serum copper levels and COPD was assessed using logistic regression to calculate the odds ratio (OR) and the 95% confidence interval (CI). Three models were considered. Model 1 was unadjusted; Model 2, adjusted for age, gender, and ethnicity; and Model 3 adjusted for all covariates. The evaluation of nonlinear relationships was conducted utilizing restricted cubic splines (RCS). Analyses of subgroups were performed to investigate possible variances among these groups. Every statistical evaluation was conducted utilizing R (version 4.3.2) and Empowerstats (version 2.0), considering a p<0.050 as statistically significant.

In MR analysis, to estimate the strength of the instrumental variables (IVs), the R^2^ for each genetic tool, the overall R^2^ and the F-statistic was calculated. For evaluating the link between serum copper levels and COPD, three MR methods were utilized: the weighted median method, MR-Egger method, and inverse variance weighting (IVW). Sensitivity analysis included Cochran’s Q statistic for IVW and Rucker’s Q statistic for MR-Egger to detect heterogeneity (p>0.050 for no heterogeneity), and MR-Egger regression for polymorphism (p>0.050 for no pleiotropy). An exception analysis was also conducted to assess the impact of individual country sample surveys on MR estimates.

## RESULTS

### Demographic characteristics

The study included 3166 US participants (2910 controls and 256 with COPD) from the 2011–2016 NHANES database. Significant differences were observed between the COPD and control groups in age, ethnicity, education level, PIR, BMI, CVD, hypertension, diabetes, and smoking habits (p<0.050). COPD patients were significantly older (≥60 years, 53.12% vs controls, 29.38%), more likely to be Non-Hispanic White (63.28% vs 38.04%), and had a lower prevalence of higher level of education (47.65% vs 57.14% with education greater than high school). The COPD group also had a higher proportion of individuals with low PIR (50.78% vs 33.23%), higher BMI (48.05% with BMI ≥30 vs 38.87%), and a greater burden of comorbidities including cardiovascular disease (30.47% vs 8.32%), hypertension (57.03% vs 33.75%), and diabetes (26.95% vs 16.53%). Additionally, a significantly higher rate of current and former smokers was observed in the COPD group (72.66% vs 40.62% in controls). Detailed effect sizes for all demographic variables are presented in Table 1.

**Table 1:** Weighted baseline characteristics of participants included in the NHANES observational study (2011–2016, N=3166). Variables are summarized to describe demographic, lifestyle, and clinical features of the study population

*Characteristics*	*Non-COPD (N=2910) n (%)*	*COPD (N=256) n (%)*	*p*
**Age** (years)			<0.001
20–39	1070 (36.77)	45 (17.58)	
40–59	985 (33.85)	75 (29.30)	
≥60	855 (29.38)	136 (53.12)	
**Gender**			0.050
Male	1436 (49.35)	110 (42.97)	
Female	1474 (50.65)	146 (57.03)	
**Race/ethnicity**			<0.001
Mexican American	458 (15.74)	18 (7.03)	
Other Hispanic	320 (11.00)	25 (9.77)	
Non-Hispanic White	1107 (38.04)	162 (63.28)	
Non-Hispanic Black	579 (19.90)	38 (14.84)	
Other race	446 (15.33)	13 (5.08)	
**Education level**			<0.001
Lower than high school	608 (20.89)	65 (25.39)	
High school	639 (21.96)	69 (26.95)	
Higher than high school	1663 (57.14)	122 (47.65)	
**PIR**			<0.001
Low (<1.40)	967 (33.23)	130 (50.78)	
Medium (1.40–3.49)	1028 (35.33)	81 (31.64)	
High (>3.49)	915 (31.44)	45 (17.58)	
**BMI** (kg/m^2^)			<0.001
<25	850 (29.21)	47 (18.36)	
25–29.9	929 (31.92)	86 (33.59)	
≥30	1131 (38.87)	123 (48.05)	
**Cardiovascular disease**			<0.001
No	2668 (91.68)	178 (69.53)	
Yes	242 (8.32)	78 (30.47)	
**Hypertension**			<0.001
No	1928 (66.25)	110 (42.97)	
Yes	982 (33.75)	146 (57.03)	
**Diabetes**			<0.001
No	2429 (83.47)	187 (73.05)	
Yes	481 (16.53)	69 (26.95)	
**Smoking status**			<0.001
Never	1728 (59.38)	70 (27.34)	
Former	660 (22.68)	89 (34.77)	
Current	522 (17.94)	97 (37.89)	
**Serum Cu** (µg/dL)			<0.001
T1 (24.7–105.7)	1008 (34.64)	46 (17.97)	
T2 (105.8–127.4)	960 (32.99)	93 (36.33)	
T3 (127.5–306.6)	942 (32.37)	117 (45.70)	

### Associations between serum copper with COPD

**Figure 2:**
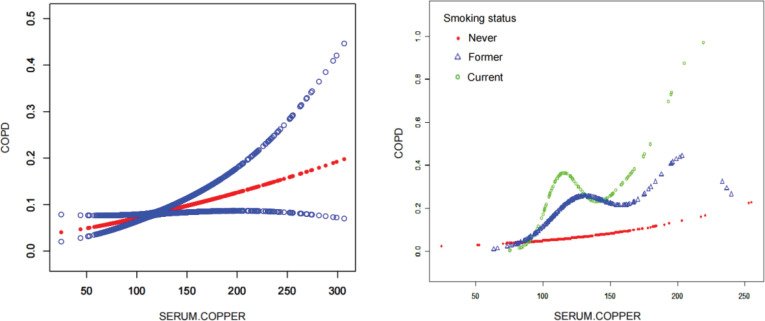
Non-linear association between serum copper and COPD in the NHANES observational study (2011–2016, N=3166): A) Restricted cubic spline analysis showing the fitted relationship (red line) with 95% confidence intervals (blue lines) in the overall population, indicating a potential non-linear trend; B) Smooth curve fitting stratified by smoking status (never, former, and current), showing distinct patterns of association between serum copper levels and COPD

Table 2 illustrates a consistent positive relationship between serum copper concentrations and COPD in every regression model. The results showed a direct relationship between elevated copper levels in the serum and the emergence of COPD in three different models. The p-trend trend=0.025 (T2, 95% CI: 1.00–2.20; T3, 95% CI: 1.09–2.49), and in the fully adjusted model, copper levels in the second and third quartiles showed OR=1.48 and OR=1.65 higher probability of COPD compared to the first quartile, respectively. The RCS analysis indicated a direct correlation between the levels of copper in the serum and the risk of COPD (Figure 2A). To further investigate whether smoking status influenced this association, we conducted stratified smooth curve fitting analyses. The smooth curve fitting results demonstrated distinct patterns according to smoking status (Figure 2B). In never smokers, serum copper levels showed a mild linear increase in relation to COPD risk. In former smokers, the curve exhibited an N-shaped trend, with COPD risk rising initially, then declining slightly, and subsequently increasing again. In current smokers, the association followed an M-shaped pattern, with COPD risk peaking at lower serum copper levels, declining thereafter, and rising again to form a second peak. These findings suggest that the relationship between serum copper and COPD is nonlinear and may be modified by smoking status.

**Table 2:** Multivariate adjusted logistic regression of COPD risk associated with serum copper in the NHANES observational study (2011–2016, N=3166)

*Variables*	*Model 1 OR (95% Cl), p*	*Model 2 OR (95% Cl), p*	*Model 3 OR (95% Cl), p*
**Serum Cu** (µg/dL)			
T1 (24.7–105.7) ®	1	1	1
T2 (105.8–127.4)	2.12 (1.47–3.06), <0.001	1.95 (1.34–2.86), 0.001	1.48 (1.00–2.20), 0.049
T3 (127.5–306.6)	2.72 (1.91–3.87), <0.0001	2.82 (1.90–4.18), <0.0001	1.65 (1.09–2.49), 0.0184
p for trend	<0.001	<0.001	0.025

Model 1: unadjusted. Model 2: partially adjusted. Model 3: fully adjusted. ® Reference category.

### Subgroup analyses

**Figure 3:**
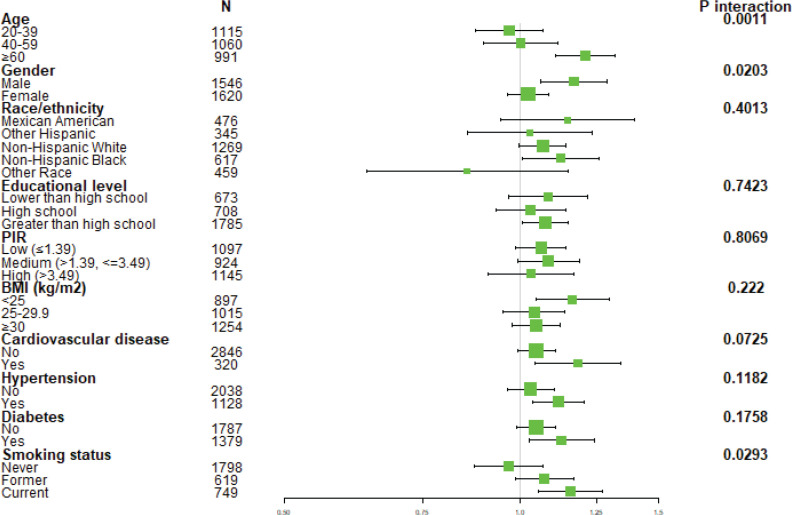
Subgroup analysis of the association between serum copper and COPD in the NHANES observational study (2011–2016, N=3166). Analyses were stratified by demographic and clinical characteristics to examine potential effect modifications

Subgroup analyses, shown in a forest plot (Figure 3), revealed inconsistent associations between serum copper levels and COPD. Significant interactions were observed for age, gender, and smoking status (p for all interactions <0.050). Remarkably, barring never smokers, individuals from various ethnic backgrounds, and those aged 20–39 years, a steady positive link was observed between the onset risks of COPD and blood copper concentrations across the sample.

### MR of serum copper and COPD and sensitivity analyses

**Figure 4:**
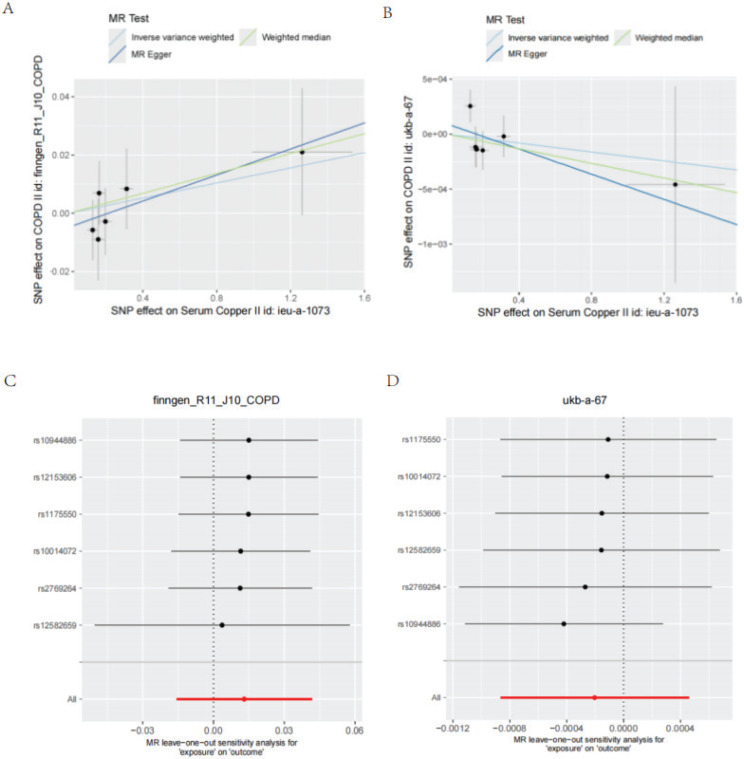
Mendelian randomization analyses evaluating the causal effect of serum copper on COPD: A, B) Scatter plots illustrating the associations between genetically predicted serum copper levels and COPD risk based on GWAS summary statistics (serum copper, N=2603; COPD, FinnGen: 21617 cases and 372627 controls; IEU, 1179 cases and 335980 controls); C, D) Leave-one-out sensitivity analysis for the effect of serum copper on COPD, demonstrating the robustness of the MR results by iteratively excluding each genetic variant

Following the verification of a positive link between serum copper levels and COPD in the observational research, a dual-sample MR analysis was conducted to evaluate potential causality. As seen in Table 3, we screened six SNPs as serum copper instrumental variables. Every SNP exhibited F-statistic values exceeding 10, signifying their robustness as tools, thereby diminishing bias in IVs calculations, refer to Supplementary file Table 2 for additional details on genetic instruments. Our MR study, utilizing three distinct approaches – IVW, MR-Egger, and weighted median – suggested no significant association between serum copper levels and the risk of COPD (p>0.050) (Table 4). Forest and scatter plots of the MR analysis are shown in Figures 4A and 4B. Our analysis of MR for diversity and horizontal pleiotropy through sensitivity tests revealed that neither the horizontal pleiotropy nor the heterogeneity held statistical significance (Supplementary file Table 3). The ‘leave-one-out’ analysis supported the robustness of the MR results, with results remaining consistent when individual SNPs were excluded (Figures 4C and 4D). These results indicated the robustness of the MR analysis.

**Table 3:** The selected SNPs associated with serum copper levels identified from genome-wide association studies (GWAS; N=2603). The table reports the chromosome (chr), position (pos), effect allele, other allele, effect size (beta), standard error (SE), and p-value for each selected SNP used as instrumental variables in MR analyses

*SNP*	*chr*	*pos*	*effect allele*	*other allele*	*β*	*SE*	*p*
rs10014072	4	113948790	G	A	-0.164	0.034	7.05×10^-7^
rs10944886	6	66926102	C	T	-0.129	0.028	2.04×10^-6^
rs1175550	1	3691528	G	A	0.198	0.032	3.06×10^-10^
rs12153606	5	84583769	T	G	-0.159	0.034	1.46×10^-6^
rs12582659	12	76064528	C	T	1.262	0.270	1.48×10^-6^
rs2769264	1	151344741	G	T	0.313	0.034	1.69×10^-20^

**Table 4:** MR analyzes results assessing the causal effect of serum copper on COPD. Analyses were performed using six genetic instruments for serum copper (IEU OpenGWAS, N=2603) and outcome data from two independent consortia (FinnGen and UK Biobank)

*Outcome ID*	*Method*	*OR*	*95% CI*	*p*
**FinnGen_R11_J10_COPD**	IVW	1.013084	0.9845025–1.042496	0.373
	MR Egger	1.022627	0.9829215–1.063937	0.330
	Weighted median	1.017235	0.9823879–1.053317	0.337
**ukb-a-67**	IVW	0.9997964	0.9991355–1.000458	0.546
	MR Egger	0.9994272	0.9979963–1.000860	0.477
	Weighted median	0.9996671	0.9988448–1.000490	0.428

## DISCUSSION

As far as we are aware, this is one of the first studies investigating the link between serum copper concentrations and COPD, initially merging a comprehensive observational study with MR analysis of genetic information. Importantly, given the central role of tobacco exposure in COPD, we also examined smoking as a key factor in our analyses. Despite the MR analysis outcomes not supporting a causal effect between serum copper concentrations and COPD, our observational research identified a positive association between them.

Research indicates that copper plays a pivotal role as a cofactor in key metabolic enzymes, participating in biological activities such as biological compound synthesis, antioxidant defense, and mitochondrial respiration^11^. Nevertheless, an overabundance of copper can also result in cytotoxicity and pathological bodily conditions. A significant demographic study in Toronto revealed that prolonged exposure to iron and copper in PM2.5, correlated with a higher incidence of COPD^23^. Studies using cross-sectional data from the 2015–2016 NHANES indicate a potential rise in the risk of obstructive lung disease among US men, due to increased serum copper levels^7^. Numerous studies have also shown elevated copper levels in the serum and sputum of COPD patients compared to those in healthy individuals^12,13,25^. These findings are consistent with our observational study that serum copper content is positively associated with COPD. Our findings reveal distinct patterns of COPD risk across smoking statuses: a mild linear increase in never smokers, an N-shaped trend in former smokers, and an M-shaped trend in current smokers. These patterns suggest a non-linear relationship between serum copper and COPD, likely modulated by smoking status.

The development of COPD is closely linked to oxidative stress^26^, tobacco smoke is known to influence metal homeostasis, including copper absorption and distribution, potentially amplifying oxidative stress in the lung. Copper can promote ROS production via a Fenton-like reaction^27^. Furthermore, high copper exposure significantly lowers glutathione, an antioxidant, leading to the destruction of lung tissue^28,29^. Elevated serum copper levels may also increase COPD risk by raising ceruloplasmin, a key acute-phase reactant in COPD^30^. Notably, tobacco smoke itself contributes to oxidative stress and may interact with copper metabolism, compounding the risk of lung injury.

Notably, in research involving animal COPD models, scientists have hypothesized a link between emphysema and the intake of copper in diets^17^. The emergence of emphysema is largely attributed to the disparity between elastase and its inhibitors^31^. The enzyme lysine oxidase, reliant on copper, plays a direct role in maintaining alveolar epithelial cells’ integrity. Its function is hindered by a lack of copper, subsequently influencing elastin production^17^. Furthermore, the study revealed that copper, found during smoking or e-cigarette usage, aids in generating NADPH^32^. Nonetheless, it is important to note that NADPH can also initiate the oxidation of DNA. Furthermore, a reverse relationship exists between the intensity of COPD and the concentration of antioxidant factors such as SOD, GSH, and GPx^33^. Consequently, to preserve standard physiological operations, it is essential to keep the copper levels in human serum within a specific spectrum. More investigation is required to completely understand the intricate association between body copper levels and the development of COPD. Both high and low serum copper levels may contribute to COPD through distinct pathways.

However, the MR analysis outcomes did not support a potential causal effect between serum copper concentrations and COPD. This implies that the previously noted link between serum copper levels and COPD in prior observational research might be influenced by different functional routes instead of a direct causal association. It is probable that the disease’s advancement is shaped by various confounding factors that accumulate over time. Serum copper elevation may be a consequence rather than cause of COPD, potentially driven by chronic inflammation altering copper metabolism, or it may act as a potentiating factor that requires the presence of a primary insult like tobacco smoke.

### Strengths and limitations

A significant strength of this study is that it draws from the US population of the NHANES database, ensuring highly representative results due to the rigorous data collection process. Additionally, MR effectively reduces confounding variables and enhances the reliability of causal inference by using genetic diversity as a key exposure variable. However, there are some limitations in the study. First, COPD diagnosis in NHANES 2013–2016 relied on self-reported physician diagnosis without spirometry confirmation, potentially introducing misclassification bias. Second, copper levels are measured in blood or blood cells, but factors influencing copper concentrations may vary across other tissues. Third, the threshold for genome-wide significance was established at p<5×10^-6^, due to the insufficiency of SNPs at this level of p<5×10^-8^ for further MR analysis. Using a more flexible p-value threshold in MR increases the number of SNPs, thereby enhancing statistical power and supporting more robust causal inference. Although this approach slightly raises the risk of false positives, the chosen threshold remains stringent enough to minimize bias, consistent with previous studies^34,35^.

Furthermore, several inherent limitations of the MR approach warrant consideration: the GWAS data we used are predominantly of European ancestry, limiting generalizability to other populations; gene–environment interactions (for example, smoking intensity, diet, or occupational exposures) were not evaluated; unobserved and horizontal pleiotropy cannot be entirely excluded and may bias estimates; potential violations of core MR assumptions (relevance, independence, exclusion restriction) could affect causal inference despite sensitivity analyses; and we lacked direct dose–response measures (e.g. pack-years or cotinine) to assess exposure gradients. Taken together, these limitations highlight that the interplay between smoking, copper, and COPD is complex. Our results should be interpreted cautiously, and future studies with more detailed smoking exposure data and stronger instruments are needed to confirm these findings and unravel the specific biological mechanisms of this interaction.

## CONCLUSIONS

Although our observational research indicates a direct link between copper levels in the serum and COPD, the existing data are inadequate to confirm a definitive causal relationship. The MR study failed to establish a cause-and-effect link, probably because of the impact of unregulated variables. Furthermore, the possible link between serum copper levels and the advancement of COPD, like sudden flare-ups, is still ambiguous, necessitating additional research.

## Supplementary Material



## Data Availability

The data supporting this research are available from the authors on reasonable request.
