# Supplementary material for: Association between serum copper and COPD: Insights from NHANES 2011–2016 and Mendelian randomization study
Source: Tob Induc Dis. 2025 Oct 17;23:10.18332/tid/210412. doi: 10.18332/tid/210412 (PMC12531986; doi:10.18332/tid/210412)
Supplement: Supplementary file 1 [file TID-23-156-s1.pdf]

SNP	Associated Trait	PMID	Effect (Beta/OR)
rs10014072	Blood trace element (Cu levels)	23720494	0.097-0.231
rs10014072	interferon-related traits	33104735	NA
rs10014072	interferon-related traits	33104735	NA
rs10014072	Total testosterone levels	32042192	0.0074-0.0139
rs10014072	Total testosterone levels	36653534	0.015-0.032
rs10944886	Gut microbial network clusters (Pink (at 1 year) x Vagina	40187613	-0.02
rs10944886	Response to anti-retroviral therapy (ddI/d4T) in HIV-1	24554482	NA
rs10944886	Blood trace element (Cu levels)	23720494	0.074-0.184
rs10944886	Exhaled nitric oxide levels	28109725	NA
rs10944886	Exhaled nitric oxide output	28109725	/
rs10944886	Endometriosis or depression (pleiotropy)	32959083	0.02-0.04
rs10944886	Major depressive disorder (MTAG)	33479212	0.018-0.035
rs10944886	Smoking initiation (ever regular vs never regular) (MTA	30643251	0.0039-0.0084
rs10944886	Obesity class II and Attention deficit hyperactivity dis	36137488	NA
rs1175550	Hemoglobin concentration	27863252	0.024-0.04
rs1175550	Hemoglobin levels	32327693	NA
rs1175550	Reticulocyte production index	37596262	0.032-0.065
rs1175550	Mean corpuscular hemoglobin concentration	27863252	0.049-0.065
rs1175550	High light scatter reticulocyte count	27863252	0.055-0.071
rs1175550	High light scatter reticulocyte percentage of red cells	27863252	0.05-0.067()
rs1175550	Reticulocyte fraction of red cells	27863252	0.044-0.06
rs1175550	Blood trace element (Cu levels)	23720494	0.14-0.26
rs1175550	Red cell distribution width	28957414	0.027-0.046
rs1175550	Immature fraction of reticulocytes	27863252	0.037-0.054
rs1175550	Reticulocyte count	27863252	0.05-0.066
rs1175550	Red blood cell count	27863252	0.022-0.038
rs1175550	Red cell distribution width	32888493	0.034-0.044
rs1175550	Red blood cell count	32888493	0.022-0.031
rs1175550	Mean corpuscular volume	32888494	0.019-0.028
rs1175550	Red blood cell count	32888493	NA
rs1175550	Red cell distribution width	32888493	NA
rs1175550	Mean corpuscular hemoglobin concentration	34594039	0.035-0.046
rs1175550	Red blood cell count	34594039	0.017-0.027
rs1175550	Red blood cell erythrocyte distribution width (UKB data	39789286	0.03-0.04
rs1175550	Reticulocyte count (UKB data field 30250)	39789286	0.038-0.048
rs1175550	Reticulocyte percentage (UKB data field 30240)	39789286	0.035-0.045
rs1175550	Red blood cell count	30595370	NA
rs1175550	Hemoglobin concentration	32888493	0.026-0.035
rs1175550	Hemoglobin concentration	32888493	NA
rs1175550	Red cell distribution width	30595370	NA
rs1175550	Mean corpuscular hemoglobin concentration	32888493	0.052-0.061
rs1175550	High light scatter reticulocyte count	32888494	0.047-0.057
rs1175550	High light scatter reticulocyte percentage of red cells	32888494	0.043-0.053
rs1175550	Hemoglobin	32888494	0.027-0.037

rs1175550	Mean corpuscular hemoglobin concentration	32888493	NA
rs1175550	Immature fraction of reticulocytes	32888494	0.03-0.04
rs1175550	Mean corpuscular hemoglobin concentration	32888494	0.05-0.06
rs1175550	Reticulocyte fraction of red cells	32888494	0.04-0.051
rs1175550	Reticulocyte count	32888494	0.045-0.055
rs1175550	Mean spheric corpuscular volume	32888494	0.045-0.055
rs1175550	Red blood cell count	32888494	0.022-0.032
rs1175550	Red cell distribution width	32888494	0.033-0.043
rs1175550	High light scatter reticulocyte percentage (UKB data fie	39789286	0.038-0.047
rs1175550	Glycated haemoglobin HbA1c levels (UKB data field 307	39789286	0.033-0.042
rs1175550	Red blood cell erythrocyte count (UKB data field 30010)	39789286	0.017-0.025
rs1175550	Mean sphered cell volume (UKB data field 30270)	39789286	0.034-0.043
rs1175550	Mean corpuscular haemoglobin concentration (UKB dat	39789286	0.033-0.043
rs1175550	Immature reticulocyte fraction (UKB data field 30280)	39789286	0.025-0.035
rs1175550	Hemoglobin	34594039	0.021-0.03
rs1175550	Hemoglobin A1c levels	34594039	0.037-0.048
rs1175550	Blood cell traits latent factor 12 (red cell)	40220762	NA
rs1175550	Blood cell traits latent factor 4 (red cell)	40220762	NA
rs1175550	Medium fluorescent percentage of reticulocytes	37596262	0.055-0.087
rs1175550	Low fluorescent percentage of reticulocytes	37596262	0.054-0.086
rs1175550	Red cell distribution width - standard deviation	37596262	0.047-0.079
rs1175550	red cell diameter width (RDW, mean, inv-norm transfo	39024449	0.031-0.045
rs1175550	mean corpuscular hemoglobin concentration (MCHC, m	39024449	0.067-0.08
rs1175550	mean corpuscular hemoglobin concentration (MCHC, m	39024449	0.072-0.086
rs1175550	mean corpuscular volume (MCV, minimum, inv-norm tr	39024449	0.027-0.042
rs1175550	mean corpuscular hemoglobin concentration (MCHC, m	39024449	0.054-0.066
rs1175550	mean corpuscular volume (MCV, maximum, inv-norm tr	39024449	0.029-0.044
rs1175550	red cell diameter width (RDW, minimum, inv-norm tran	39024449	0.031-0.045
rs1175550	mean corpuscular volume (MCV, mean, inv-norm trans	39024449	0.03-0.045
rs1175550	mean corpuscular hemoglobin concentration (MCHC, m	39024449	0.096-0.109
rs1175550	mean corpuscular hemoglobin concentration (MCHC, m	39024449	0.11-0.12
rs1175550	mean corpuscular hemoglobin concentration (MCHC, m	39024449	0.079-0.093
rs1175550	mean corpuscular hemoglobin concentration (MCHC, m	39024449	0.027-0.047
rs1175550	Hemoglobin A1c (HbA1c, mean, inv-norm transformed)	39024449	0.022-0.037
rs1175550	Hemoglobin A1c (HbA1c, minimum, inv-norm transform	39024449	0.023-0.037
rs1175550	Hemoglobin A1c (HbA1c, maximum, inv-norm transfor	39024449	0.019-0.034
rs1175550	red cell diameter width (RDW, maximum, inv-norm tra	39024449	0.021-0.033
rs1175550	red cell diameter width (RDW, minimum, inv-norm tran	39024449	0.031-0.045
rs1175550	red cell diameter width (RDW, mean, inv-norm transfo	39024449	0.028-0.042
rs1175550	Glycated hemoglobin levels	34059833	0.0069-0.0127
rs1175550	High fluorescent percentage of reticulocytes	37596262	0.036-0.069
rs1175550	Hematocrit	32888494	0.0096-0.0187
rs1175550	Blood cell traits latent factor 14 (red cell)	40220762	NA
rs1175550	Reticulocyte side fluorescence	37596262	0.049-0.082
rs1175550	Blood cell traits latent factor 12 (red cell)	40220762	NA

rs1175550	Glycated hemoglobin levels	33462484	0.031-0.042
rs1175550	Red blood cell side fluorescence	37596262	0.036-0.072
rs1175550	Ovarian cancer	32887889	NA
rs1175550	Red cell distribution width	27863252	0.03-0.046
rs1175550	Blood cell traits latent factor 4 (red cell)	40220762	NA
rs1175550	Blood cell traits latent factor 14 (red cell)	40220762	NA
rs1175550	Hemoglobin concentration	35964923	NA
rs1175550	Mean corpuscular volume	32888493	NA
rs1175550	Mean corpuscular volume	27863252	0.022-0.038
rs1175550	Mean corpuscular volume	32888493	0.02-0.028
rs1175550	hemoglobin (maximum, inv-norm transformed)	39024449	0.015-0.027
rs1175550	Blood cell traits latent factor 4 (red cell)	40220762	NA
rs1175550	Blood cell traits latent factor 12 (red cell)	40220762	NA
rs1175550	Blood cell traits latent factor 14 (red cell)	40220762	NA
rs12153606	Blood trace element (Cu levels)	23720494	0.092-0.226
rs12153606	Adolescent idiopathic scoliosis	30019117	NA
rs12153606	Gut microbiota relative abundance (Eubacterium belon	33208821	0.64-1.62
rs12582659	Blood trace element (Cu levels)	23720494	0.73-1.79
rs2769264	Blood trace element (Cu levels)	23720494	0.25-0.38
rs2769264	Protein quantitative trait loci (liver)	32778093	NA
rs2769264	Body mass index	38538606	0.011-0.023
rs2769264	Blood pressure (pleiotropy model 1 DBP adjusted for es	34989438	0.038-0.142
rs2769264	Blood pressure (pleiotropy model 2 SBP adjusted for es	34989438	0.07-0.209
rs2769264	Telomere length (principal component 1)	39192095	0.018-0.029
rs2769264	Depression (broad)	29662059	0.0053-0.0115
rs2769264	Educational attainment	35361970	0.0058-0.0124
rs2769264	Protein quantitative trait loci (liver)	32778093	NA
rs2769264	Proteasome subunit beta type-4 levels	39528825	0.93-1.05
rs2769264	Proteasome subunit beta type-4 levels	34648354	0.46-0.53
rs2769264	Protein quantitative trait loci (liver)	32778093	NA
rs2769264	Protein quantitative trait loci (liver)	32778093	NA
rs2769264	Longevity	31484785	0.0073-0.0179
rs2769264	Educational attainment	35361970	0.0057-0.0111

p
1.00E-06
3.00E-06
3.00E-06
2.00E-10
3.00E-08
7.00E-10
6.00E-06
4.00E-06
2.00E-06
7.00E-07
2.00E-09
1.00E-09
6.00E-08
1.00E-08
2.00E-14
1.00E-28
6.00E-09
1.00E-43
1.00E-49
2.00E-43
3.00E-34
5.00E-10
2.00E-16
3.00E-27
2.00E-42
9.00E-13
3.00E-61
2.00E-30
7.00E-20
2.00E-28
4.00E-61
1.00E-45
3.00E-19
6.00E-52
3.00E-78
4.00E-66
2.00E-30
1.00E-40
4.00E-38
2.00E-63
5.00E-123
8.00E-85
5.00E-72
2.00E-33

5.00E-119
1.00E-38
8.00E-93
1.00E-64
9.00E-79
6.00E-80
3.00E-24
4.00E-46
8.00E-76
8.00E-69
2.00E-26
3.00E-63
3.00E-48
4.00E-35
2.00E-28
2.00E-52
1.00E-14
2.00E-11
2.00E-17
5.00E-17
2.00E-14
9.00E-27
3.00E-105
5.00E-117
4.00E-19
1.00E-77
6.00E-21
6.00E-27
3.00E-22
1.00E-198
1.00E-234
5.00E-139
3.00E-13
3.00E-14
3.00E-15
3.00E-12
2.00E-16
6.00E-26
3.00E-23
7.00E-13
3.00E-10
9.00E-10
3.00E-08
7.00E-15
3.00E-09

2.00E-34
4.00E-09
6.00E-08
7.00E-21
3.00E-09
3.00E-12
2.00E-08
4.00E-29
1.00E-13
8.00E-31
1.00E-11
1.00E-08
3.00E-09
1.00E-08
2.00E-06
4.00E-06
7.00E-06
3.00E-06
3.00E-20
6.00E-12
3.00E-08
2.00E-08
1.00E-08
1.00E-17
2.00E-07
6.00E-08
1.00E-12
3.00E-207
1.00E-186
2.00E-16
1.00E-10
3.00E-06
2.00E-09

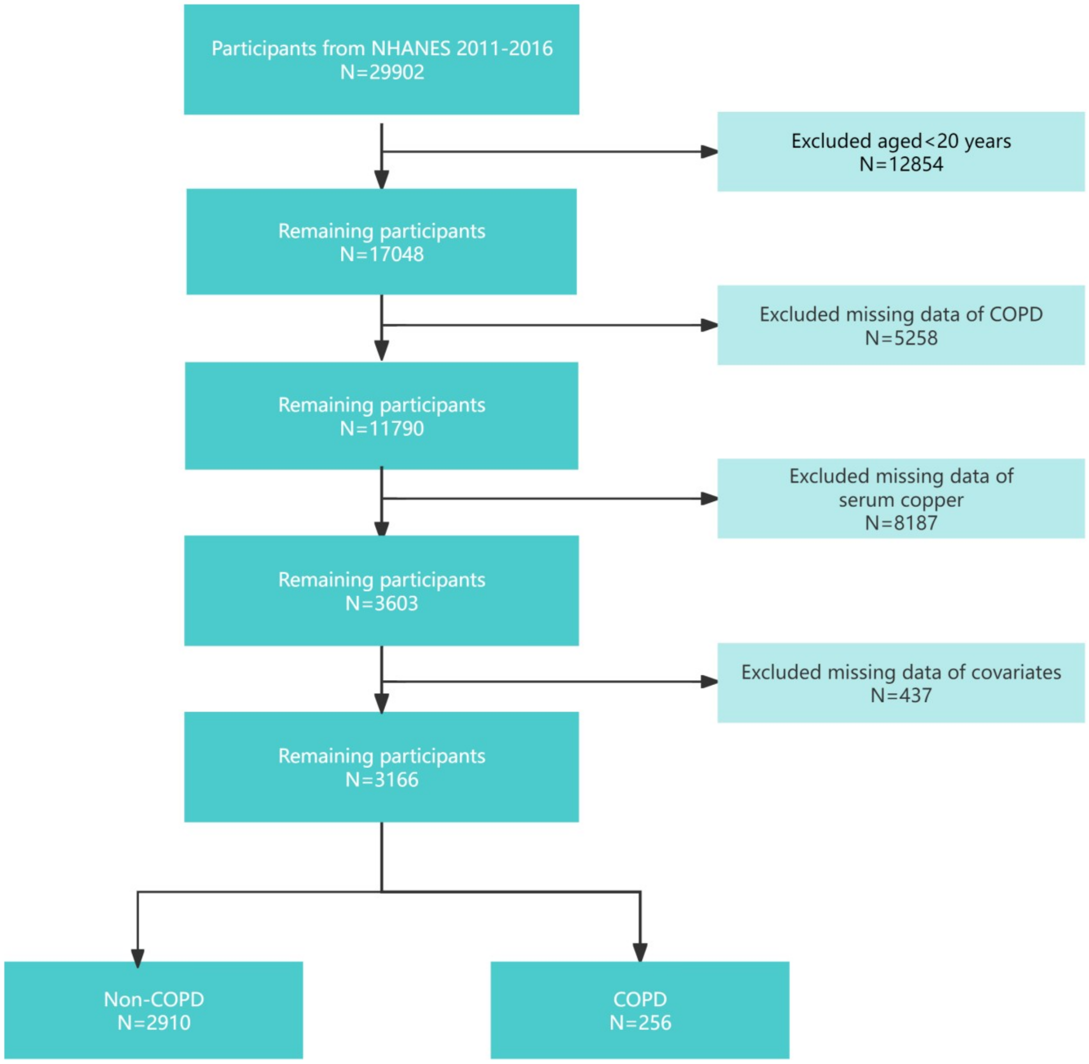

Supplementary Figure 1 Flow chart of participant selection from NHANES 2011–2016.

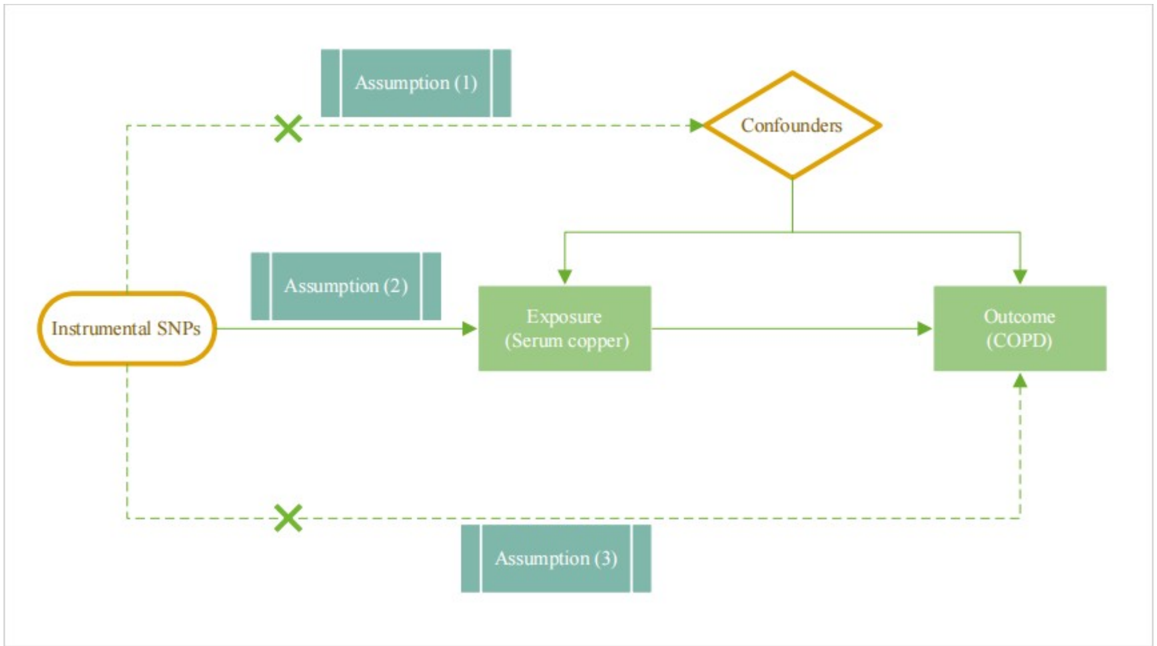

Supplementary Figure 2 Conceptual framework of the three fundamental assumptions of two-sample MR.

Supplementary Table 1 Characteristics of GWAS datasets used as genetic instruments, including data source, sample size, ethnicity, and web source

Data	Data source	Sample size	Ethnicity	Web source
Serum copper	IEU	2603	European	https://gwas.mrcieu.ac.uk
COPD	Finngen	21617/372627	European	https://www.finngen.fi/en/access_results
COPD	UKBB	1179/335980	Europea	http://www.nealelab.is/uk-

Information Classification: General

	n	biobank

Supplementary Table 2 Information of genetic instruments, with SNP-level R^2 values and F-statistics

SNP	R^2	F-statistic
rs10014072	8.86×10^{-3}	23.25
rs10944886	8.09×10^{-3}	21.21
rs1175550	1.45×10^{-2}	38.26
rs12153606	8.33×10^{-3}	21.85
rs12582659	8.32×10^{-3}	21.83
	overall R^2: 0.0796	

Supplementary Table 3 Sensitivity analysis for the effect of serum copper on COPD using MR-Egger and IVW approaches, including heterogeneity (Cochran's Q) and pleiotropy tests

Outcome ID	Method	Value	*p*
finngen_R11_J10_COPD	MR Egger (heterogeneity)	Cochran's Q = 1.244	0.871
	IVW (heterogeneity)	Cochran's Q = 1.695	0.890
	MR Egger (pleiotropy)	Intercept = -4.782 × 10^{-3}	0.539
ukb-a-67	MR Egger (heterogeneity)	Cochran's Q = 4.539	0.338

Information Classification: General

	IVW (heterogeneity)	Cochran's Q = 4.922	0.426
	MR Egger (pleiotropy)	Intercept = 9.346 × 10^{-5}	0.593

© 2025 Qu J. et al.

Information Classification: General
